# The Effects of Stakeholder Perceptions on the Use of Humanoid Robots in Care for Older Adults: Postinteraction Cross-Sectional Study

**DOI:** 10.2196/46617

**Published:** 2023-08-04

**Authors:** Slawomir Tobis, Joanna Piasek-Skupna, Agnieszka Neumann-Podczaska, Aleksandra Suwalska, Katarzyna Wieczorowska-Tobis

**Affiliations:** 1 Department of Occupational Therapy Poznan University of Medical Sciences Poznan Poland; 2 Institute of Robotics and Machine Intelligence Poznan University of Technology Poznan Poland; 3 Department of Palliative Medicine Poznan University of Medical Sciences Poznan Poland; 4 Department of Mental Health, Chair of Psychiatry Poznan University of Medical Sciences Poznan Poland

**Keywords:** older adult, care robot, stakeholder, perception, needs and requirements, user need, patient need, elder, gerontology, geriatric, caregiver, attitude, opinion, home care, caregiving, robot

## Abstract

**Background:**

Efficient use of humanoid social robots in the care for older adults requires precise knowledge of expectations in this area. There is little research in this field that includes the interaction of stakeholders with the robot. Even fewer studies have compared the perceptions of older people (as care recipients) and professional caregivers (representing those taking care of older adults in teams with robots).

**Objective:**

The aim of this study was to analyze whether specific aspects of the perceptions about humanoid robots influence attitudes after interacting with the robot and to compare the opinions of different stakeholders (older people and their professional caregivers) on this topic. We analyzed the potential impact of the differences in perception of the robot between stakeholder groups with respect to how the robot should be designed and tailored to fit the specific needs of future users. We also attempted to define areas where targeted educational activities could bring the attitudes of the two groups of stakeholders closer to each other.

**Methods:**

The studied group was a conveniently available sample of individuals who took part in the presentation of and interaction with a humanoid social robot. Among them, there were 48 community-dwelling older adults (aged ≥60 years), who were participants of day care units (which may signal the presence of self-care needs), and 53 professional caregivers. The participants were asked to express their views after an interaction with a humanoid social robot (TIAGo) using the Users’ Needs, Requirements and Abilities Questionnaire (UNRAQ) and the Godspeed Questionnaire Series (GQS).

**Results:**

Compared to the caregivers, older adults not only assessed the robot more positively with respect to its roles as a companion and assistant (*P*=.009 and *P*=.003, respectively) but also had higher scores on their need to increase their knowledge about the robot (*P*=.049). Regarding the robot’s functions, the greatest differences between groups were observed for the social aspects on the UNRAQ, including decreasing the sense of loneliness (*P*=.003) and accompanying the user in everyday activities (*P*=.005). As for the GQS, the mean scores of the Animacy, Likeability, and Perceived Intelligence scales were significantly higher for older participants than for caregivers (*P*=.04, *P*<.001, and *P*<.001, respectively). The only parameter for which the caregivers’ scores were higher than those of the older adults was the Artificial-Lifelike item from the Anthropomorphism scale of the GQS (*P*=.03).

**Conclusions:**

The acceptance of the social functions of a humanoid robot is related to its perception in all analyzed aspects, whereas the expected usefulness of a care robot is not linked to aspects of anthropomorphism. Successful implementation of robots in the care for older people thus depends on considering not only the fears, needs, and requirements of various stakeholders but also on the perceptions of the robot. Given the differences between the stakeholders, targeted and properly structured educational and training activities for caregivers and prospective users may enable a seamless integration of robotic technologies in care provision.

## Introduction

The introduction of robots in the care for older people is a complex task [1]. Besides purely technological challenges, other vital aspects need to be taken into account when considering using robots in care, including a proper, well-prepared, and sufficiently timed introduction while observing ethical issues and reservations. Studying the abilities and requirements of potential robot users is thus essential; the failure of many telehealth projects could be at least partially attributed to the lack of proper assessment of the needs of future technology users and the immediate environment [2,3]. The results and conclusions of such studies have value, among others, in delivering premises for designing new technologies and devices. Don Norman (an 83-year-old author of the industry bible *Design of Everyday Things* and a former Apple vice president) stated that “the world seems designed against the elderly” [4]. As demonstrated by Mikus [5], numerous technologies that had been considered successful failed when confronted with older users. The identified reason for this phenomenon was not—as commonly believed—that older adults were not interested or able to cope with technology but rather that they had not been engaged in the design process. In a qualitative study involving various stakeholders, Peek et al [6] reported that all groups noted that new technologies should provide tangible benefits to older users. At the same time, older participants observed that technologies offering too many benefits could make their users lose independence. It is thus essential that robot designers have precise knowledge about the abilities, requirements, and expectations of future users, as only in this way can new technologies play an adequate role in implementing the paradigm of aging in place.

Factors contributing to the acceptance of automated/robotic helpers have been studied widely, including commercial relationships [7,8], cognitively demanding tasks [9], and children’s attitudes [10]. Nevertheless, in their recent scoping review of the acceptability of social robots, David et al [11] stated that the most frequently studied subjects were older people (32.56% of analyzed studies) and health professionals (16.28% of analyzed studies) [11].

The introduction of social robots in the care for older people requires not only their acceptance by older adults but also by their caregivers, including professional caregivers. A literature review by Zaman et al [12] showed that one-third of health care providers expressed concerns regarding the widespread use of information and communication technology interventions replacing traditional health care delivery models that could result in job loss, which has been grossly contradicted by Chang et al [13] who argued that a robot will not replace humans but rather optimize their time budget. Liao et al [14] found real-world benefits as key drivers in forming positive attitudes toward robots in health care. The results of a Danish study performed on a nationally representative group of nursing staff (employed in various health care institutions) indicated that “a prerequisite for the successful introduction of new technologies is to analyze determinants that may impede or enhance the introduction among potential users” [15]. Furthermore, Hoppe et al [16] emphasized that the cocreation of assistive robots is structurally distinct from other cocreation processes since it should involve multiple stakeholders (ie, potential users, caregivers, relatives). In addition, factors not typically included in technology acceptance models (such as hedonic and social factors) must be taken into consideration to enable successful implementation.

Our previous studies concentrated on the validation of the Users’ Needs, Requirements and Abilities Questionnaire (UNRAQ) as an assessment tool [17] and the investigation of the impact of a real-world robotic experience on the opinions related to the use of robots in care for older adults [18]. We demonstrated that the possibility of a palpable interaction with a humanoid social robot changed the perspective and had an effect on some of the studied traits of opinions toward the use of robots in care. We also previously analyzed the views of future professional caregivers (nursing and medical students) on the role of robots in the care of older people [19].

To expand on this previous work, in this study, we compared the opinions of two groups of stakeholders on the use of humanoid social robots in the care of older adults: older people themselves and their professional caregivers (those actively engaged in care). We concentrated the analysis on the potential impact of the differences in perceptions of a robot between stakeholders on the way the robot should be designed and tailored to fit the specific needs of future users. We also attempted to define areas where targeted educational activities could bring the stakeholders’ attitudes closer to each other. The aim of the study was thus to analyze whether specific aspects of the perceptions of such a robot influence the attitudes expressed after interacting with the robot.

## Methods

### Design

The participants were asked to express their views after a sufficiently timed interaction with a humanoid social robot (TIAGo, PAL Robotics, Barcelona, Spain). The opinions were collected using the previously presented UNRAQ (see Multimedia Appendix 1), which has been demonstrated to have good psychometric properties [17]. In addition, the users’ perceptions of the robot were measured with the Godspeed Questionnaire Series (GQS), which analyzes features including anthropomorphism, animacy, likeability, perceived intelligence, and perceived safety of robots [20].

### Ethical Considerations

The study protocol was verified by the Bioethics Committee of Poznan University of Medical Sciences, Poznan, Poland (protocol 711/18). All participants, after receiving a full explanation of the nature of the study and the possibility of withdrawal at any time, expressed their consent for participation. No participant decided to withdraw from the study. Gathered data were deidentified; tables built for analysis used sequentially generated ID numbers. The participants received no compensation for their participation.

### Recruitment

The study group was a conveniently available sample of individuals who took part in the presentation of and interaction with the humanoid social robot, including community-dwelling older adults (aged ≥60 years), who were participants of day care units (which may signal the presence of needs in the area of self-care), as well as professional caregivers (including students of a 1-year postgraduate course dedicated to geriatrics and care for older people during the last month of their study and final-year undergraduate students pursing a bachelor degree in occupational therapy at Poznan University of Medical Sciences). The recruitment took place at the day care units and the university. All individuals who expressed interest in participation and met the age/professional role criteria were included in the study.

### Procedure

Sociodemographic data (age, gender, marital status, living arrangement, education) were collected from all participants, along with declarative statements related to the ease of use of technological devices and self-assessments of loneliness, health, and fitness. Higher education was defined as university level/academic and ease of use of technological devices was a subjective measure in the broad sense (thus, it was not restricted to particular technologies).

The older adults and caregivers separately participated in presentations in groups of 12-20 people, followed by subsequent interaction with the machine. The interaction lasted until all participants in the group felt they had been given sufficient time to interact (actual times ranged from approximately 60 to 90 minutes). During the presentation/interaction sessions, a customized version of the TIAGo robot was used, which was wirelessly networked and equipped with a range of sensors and cameras [18] as well as a microphone, loudspeaker, and touch tablet for communication with the human. The first part of the presentation was static; the participants were then free to make use of the navigation capabilities of the robot (either semiautonomously, based on a prerecorded environment map, or directly by the user). The robot was able to provide current news and weather and display a variety of environmental values (including air temperature, pressure, and humidity) from its own or distant (networked) sensors. Other available options included cognitive games, physical exercises, dietary recommendations, reminders, and safety measures (eg, detection of unlocked doors). Finally, the participants filled out the UNRAQ to express their needs and requirements of a social robot intended to be used in care for older people and expressed their perceptions of the robot by means of the GQS.

The UNRAQ uses a 5-point Likert scale, assigning the individual answers numbers according to the following scheme: 1, I strongly disagree; 2, I partially disagree; 3, I neither agree nor disagree; 4, I partially agree; and 5, I strongly agree. This structure enables the calculation of means and SDs and is well-suited for further statistical analyses. Each of the UNRAQ’s statements can be complemented with a free-text remark. The UNRAQ has four main parts: A, Interaction of the robot and technical issues; B, Assistive role of the robot; C, Social aspects of using the robot; and D, Ethical issues. Part B is composed of 13 statements and part C comprises six statements (see Multimedia Appendix 1). For each of these areas, mean values characterizing the opinion of each participant were additionally calculated. The scheme has been previously presented in detail [18].

The GQS consists of five scales (Anthropomorphism, Animacy, Likeability, Perceived Intelligence, and Perceived Safety). Each of these scales has five items (except Perceived Safety, which has three items), also scored on a 1-5 scale. This tool has been widely used in research related to human-robot interactions [21]. We thus selected the GQS for our study since it clearly complements the UNRAQ in the evaluation of perceptions of a social robot.

### Statistical Analysis

Statistical analysis was performed with STATISTICA 13 software (TIBCO Software, Poland). Due to the relatively small sample sizes, nonparametric tests were used in further analyses. For ordinal data, the comparison between the two stakeholder groups was made with the Mann–Whitney *U* test, whereas differences in the distribution of qualitative variables between the two groups were assessed with the *χ*^2^ test with Yates correction due to a small sample size. Additionally, for numerical data, the Spearman coefficient was used as a measure of correlation.

Statistical significance was considered at *P*<.05; .05≤*P*<.10 constituted an insignificant trend.

## Results

### Study Group

A total of 101 people took part in the study, including 53 caregivers (mean age 38.6, SD 15.2 years) and 48 older people (mean age 75.5, SD 8.7 years). The caregivers were significantly younger (*P*<.001), and they also differed from the older adult group in terms of sex (higher proportion of females, *P*=.01), education (higher proportion with advanced education, *P*<.001), and partnerships (higher proportion in relationships, *P*<.001). Caregivers also less frequently declared loneliness (*P*<.001) and reported better health (*P*<.001) as well as better fitness (*P*<.001) (Table 1).

With respect to the declared ease of technology use, the statistical analysis showed a borderline value (*P*=.08) when comparing means of the self-scores on a scale of 1-5 (Table 1). Nevertheless, older adults more frequently scored negatively, with 13/48 providing scores of 1 or 2 on the Likert scale compared to only 1/53 caretakers providing low scores (*P*<.001).

**Table 1:** Detailed characteristics of the study participants.

Characteristic		Total (N=101)		Caregivers (n=53)		Older adults (n=48)		*P* value	
**Sex, n (%)**									.01
	Female	82 (81.2)	48 (90.6)		34 (70.8)				
	Male	19 (18.8)	5 (9.4)		14 (29.2)				
**Education level, n (%)**									<.001
	Below university	52 (53.1)	16 (31.4)		36 (76.6)				
	University and higher	46 (46.9)	35 (68.6)		11 (23.4)				
**Marital status, n (%)**									<.001
	Single	66 (66.0)	25 (48.1)		41 (85.4)				
	Married	34 (34.0)	27 (51.9)		7 (14.6)				
**Living arrangement, n (%)**									.58
	Alone	11 (18.0)	9 (17.0)		2 (25.0)				
	With others	50 (82.0)	44 (83.0)		6 (75.0)				
Ease of use of technology, mean (SD)		3.9 (1.2)		4.2 (0.8)		3.5 (1.5)		.08	
Self-assessment of loneliness, mean (SD)		2.2 (1.5)		1.8 (1.2)		2.8 (1.5)		<.001	
Self-assessment of health, mean (SD)		3.7 (0.9)		4.1 (0.8)		3.3 (0.8)		<.001	
Self-assessment of fitness, mean (SD)		3.8 (0.9)		4.3 (0.7)		3.3 (0.8)		<.001	

### Opinions of the Whole Study Group About a Robot in Care for Older Adults

Based on the UNRAQ, good acceptance of the robot was observed among the respondents (Table 2). As far as the GQS results are concerned, the highest scores were given by the participants in the Likeability and Perceived Intelligence scales (mean scores for all items in these two series were above 3.0 on the Likert scale).

A positive correlation was observed between the average opinion on the assistive role of the robot and the scores for all GQS scales, except Anthropomorphism (Animacy: *r*=0.2110, *P*=.04; Likeability: *r*=0.3055, *P*=.002; Perceived Intelligence: *r*=0.3052, *P*=.002; Perceived Safety: *r*=0.3543, *P*<.001). All correlations were weak to moderate. It is worth noting that the highest Spearman coefficient was observed for Perceived Safety.

For the mean values of social aspects of using the robot, relationships were found with all ranges of the GQS (Anthropomorphism: *r*=0.2443, *P*=.01; Animacy: *r*=0.2024, *P*=.04; Likeability: *r*=0.3783, *P*<.001; Perceived Intelligence: *r*=0.3547, *P*<.001; Perceived Safety: *r*=0.4652, *P*<.001). Again, all correlations were weak to moderate and the highest Spearman coefficient was found for Perceived Safety.

**Table 2:** Detailed results of the Users’ Needs, Requirements and Abilities Questionnaire.

Area of the questionnaire			Total, mean (SD)		Caregivers, mean (SD)		Older adults, mean (SD)		*P* value
**A: Interaction of the robot and technical issues**									
	A1 The robot should be a companion of the older person	3.7 (1.3)		3.6 (1.0)		3.9 (1.5)		.009	
	A2 The robot should be an assistant of the older person	4.1 (1.1)		4.0 (1.0)		4.4 (1.2)		.003	
	A3 The robot should be a useful device of the older person (something to be used when needed, with no other interaction)	4.4 (0.9)		4.3 (0.9)		4.5 (1.0)		.15	
	A4 Older adults are prepared to interact with a robot	2.2 (1.1)		2.2 (1.0)		2.3 (1.3)		.86	
	A5 Older adults are able to manage with the robot	2.6 (1.3)		2.7 (1.1)		2.5 (1.4)		.39	
	A6 Older adults want to increase their knowledge about robots to be able to operate them	3.5 (1.2)		3.3 (1.0)		3.7 (1.4)		.049	
	A7 The robot should instruct the older person what to do in case of a problem with its operation	4.6 (0.8)		4.4 (0.8)		4.8 (0.7)		.006	
	A8 The robot should be customizable (adjusted to individual user preferences and needs)	4.7 (0.7)		4.7 (0.5)		4.6 (0.8)		.82	
	A9 Older adults should be able to choose the functions of the robot they want to use and disable other ones	4.4 (0.8)		4.2 (0.8)		4.6 (0.8)		.003	
	A10 If the robot has been switched off by the owner, it should reactivate automatically (after a specific period), so that it is not forgotten in off mode	4.4 (0.9)		4.2 (0.9)		4.6 (0.9)		.004	
**B: Assistive role of the robot**									
	B1 The robot should increase the safety of the older adult’s home (eg, locking doors, detecting leaking gas)	4.8 (0.6)		4.7 (0.5)		4.9 (0.6)		.001	
	B2 The robot should help the older adult preserve their memory function (eg, by playing memory games with them)	4.6 (0.7)		4.6 (0.5)		4.7 (0.9)		.07	
	B3 The robot should encourage and guide older adults to perform physical exercises	4.6 (0.8)		4.5 (0.7)		4.7 (0.8)		.06	
	B4 The robot should provide advice about a healthy diet	4.3 (1.0)		4.2 (0.9)		4.4 (1.0)		.06	
	B5 The robot should monitor the environment (temperature, humidity) and suggest air conditioning adjustment or windows opening	4.5 (0.9)		4.5 (0.7)		4.6 (1.0)		.15	
	B6 The robot should measure the physiological parameters (blood pressure, heart rate, body temperature) of the older person	4.6 (0.9)		4.5 (0.6)		4.7 (1.1)		.002	
	B7 The robot should monitor the amount of food and fluid intake of the owner	4.2 (1.0)		4.2 (0.9)		4.3 (1.1)		.45	
	B8 The robot should remind older adults about appointments	4.5 (0.8)		4.5 (0.6)		4.4 (1.0)		.38	
	B9 The robot should remind older adults about medication	4.6 (0.7)		4.6 (0.6)		4.6 (0.9)		.30	
	B10 The robot should remind older adults about meal times and drinking	4.3 (1.1)		4.5 (0.8)		4.1 (1.3)		.30	
	B11 The robot should observe the behavior of the older person to detect falls or changes due to illness	4.6 (0.7)		4.5 (0.6)		4.7 (0.7)		.03	
	B12 The robot should call the center in case of emergency	4.9 (0.5)		4.8 (0.4)		4.9 (0.6)		.11	
	B13 The robot should help the owner find lost objects (eg, glasses, keys)	4.7 (0.6)		4.6 (0.6)		4.8 (0.7)		.04	
**C: Social aspects of using the robot**									
	C1 The robot could decrease the sense of loneliness and improve the mood of the older person	4.2 (1.0)		4.0 (0.9)		4.4 (1.0)		.003	
	C2 The robot could encourage older adults to enhance their contacts with friends	4.2 (0.9)		4.1 (0.8)		4.3 (1.0)		.05	
	C3 The robot should initiate contacts with others (calling friends, initiating Skype conversations)	4.4 (0.7)		4.2 (0.7)		4.6 (0.8)		<.001	
	C4 The robot should have entertainment functions (eg, gaming partner, reading aloud, or playing music function)	4.4 (0.8)		4.5 (0.6)		4.4 (1.0)		.29	
	C5 The robot should detect the owner’s mood (facial expression)	4.1 (1.1)		3.9 (1.0)		4.3 (1.1)		.01	
	C6 The robot should accompany the owner in everyday activities (watching TV, preparing meals)	3.8 (1.2)		3.6 (1.1)		4.1 (1.3)		.005	
**D: Ethical issues**									
	D1 The older person should have control over the robot	4.4 (0.9)		4.2 (0.8)		4.6 (0.9)		.003	
	D2 The older person should be able to send the robot to its place/docking station and keep it there	4.4 (0.9)		4.3 (0.8)		4.5 (1.0)		.14	
	D3 It is acceptable that the robot informs a family member or caregiver about the older person’s behavior/health problems	4.5 (0.7)		4.4 (0.7)		4.7 (0.7)		<.003	
	D4 The older person should be able to switch off the robot in specific situations (eg, friends’ visits, privacy reasons)	4.4 (1.0)		4.3 (0.9)		4.5 (1.1)		.01	
	D5 It is acceptable that the robot will obtain substantial information about the user (social, medical, others)	4.1 (1.1)		4.0 (1.0)		4.2 (1.2)		.03	

### Comparison of Opinions of Stakeholders (Older Individuals Living in the Community and Professional Caregivers) About a Robot in Care for Older Adults

The results of the UNRAQ showed significant differences between the views of caregivers and older adults about the role of the robot in the care of older adults. The latter group assessed the robot better in the roles of companion (*P*=.009) and assistant (*P*=.003). The older adults also provided significantly higher ratings for the statements A6 (*Older adults want to increase their knowledge about the robots to be able to operate them*, *P*=.049) and A7 (*The robot should instruct the older person what to do in case of a problem with its operation*, *P*=.006). As for the opinions on the role of the robot in 13 assistive functions, differences were observed for four statements only; in all cases, the views of older people were more positive than those of the caregivers. This included increasing the safety of the home (B1, *P*=.001), measuring physiological parameters (B6, *P*=.002), the need to observe the behavior of the older person to detect falls or changes related to illness (B11, *P*=.03), and help in finding lost items (B13, *P*=.04). However, there was no significant difference in the mean value for assistive functions between the groups (caregivers: 4.5, SD 0.4; older adults: 4.6, SD 0.7).

Opinions on social aspects of using the robot differed for 4 out of 6 statements, in which higher scores were given by the older participants than by the caregivers in all cases: C1 (*The robot could decrease the sense of loneliness and improve the mood of the older person*, *P*=.003), C3 (*The robot should initiate contacts with others*, *P*<.001), C5 (*The robot should detect the owner’s mood*, *P*=.01), and C6 (*The robot should accompany the owner in everyday activities*, *P*=.005). This translated into higher mean scores of functions for this area (older adults: 4.4, SD 0.8; caregivers: 4.0, SD 0.7; *P*=.004).

In terms of ethical issues, all statements except D2 (*The older person should be able to send the robot to its place/docking station and keep it there*) were rated significantly higher by older participants (Table 2).

**Figure 1:**
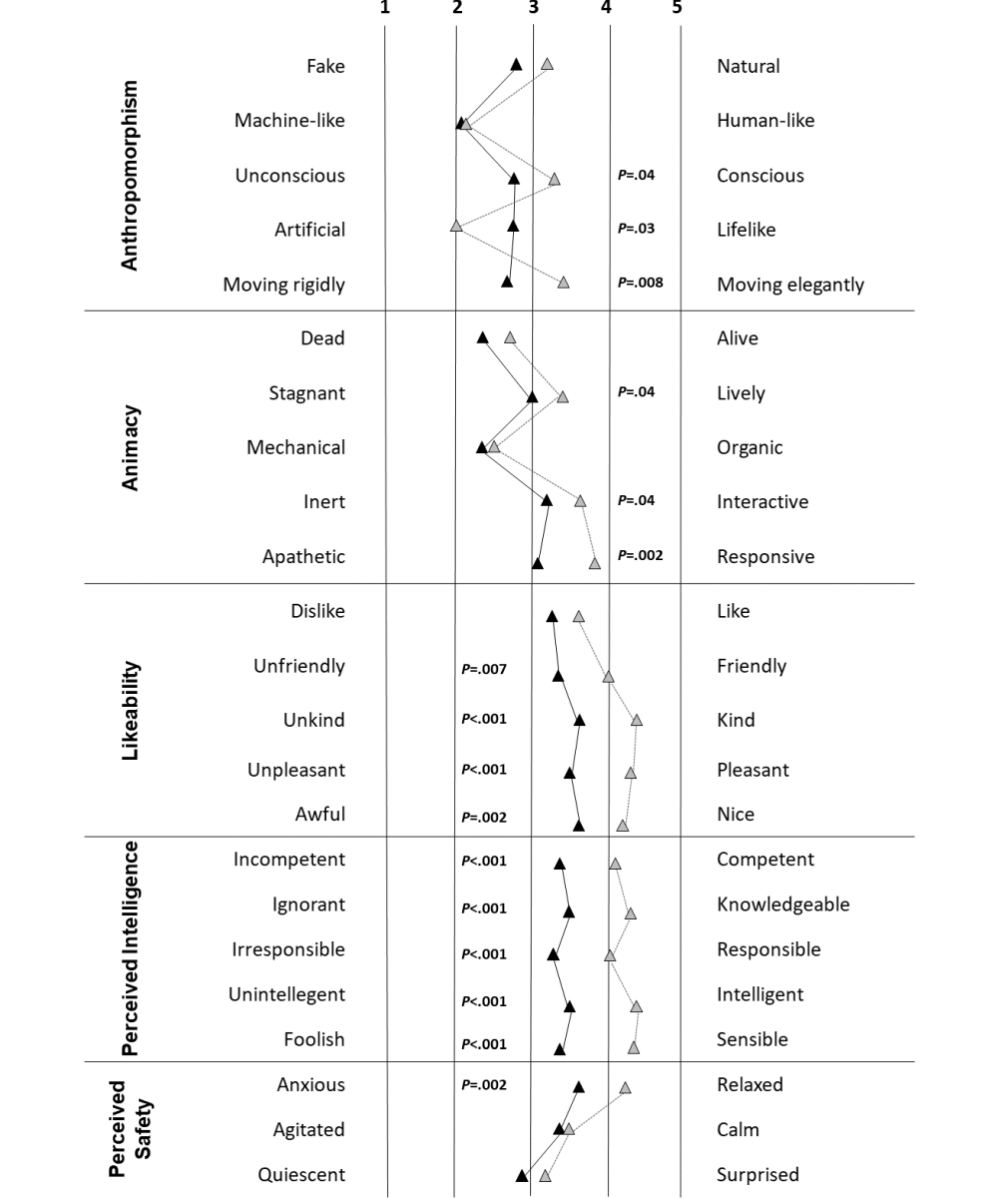
The results of the Godspeed Questionnaire Series for caregivers (solid lines) and older adults (dotted lines).

Similar to the UNRAQ, the results of the GQS differed significantly between the groups. The mean scores of the Animacy (3.2, SD 1.1 vs 2.8 SD 0.9; *P*=.04), Likeability (4.1, SD 1.0 vs 3.5, SD 1.0; *P*<.001), and Perceived Intelligence (4.2, SD 0.8 vs 3.4, SD 0.7; *P*<.001) series were significantly higher for older participants than for caregivers. For all parameters of the Anthropomorphism and Perceived Intelligence series, the opinions of older participants were decidedly more positive. An analogous relationship was also observed for selected parameters of the remaining scales (Figure 1). The only parameter for which the caregivers’ scores were higher was the Artificial-Lifelike item from the Anthropomorphism scale (*P*=.03).

## Discussion

### Principal Findings

In this study, we observed that the acceptance of social functions improved with increasing positive perception of the robot in all analyzed aspects: Anthropomorphism, Animacy, Likeability, Perceived Intelligence, and Perceived Safety. The perception of the robot should thus be considered a crucial factor for the implementation of social functions in the care context. For the assistive functions of the robot, only Anthropomorphism was found to be statistically insignificant. Both of these observations bear importance and should be taken into account when designing the social and assistive components of a robot, respectively, as it has so far not been demonstrated in this manner. de Graaf and Allouch [22] showed that a high rating of Anthropomorphism might be associated with high social acceptability and treated as a predicting variable for companionship; anthropomorphized robots are likely to be viewed as “genuine.” However, one should also bear in mind the potential of the uncanny valley phenomenon in this case [23].

### Comparison to Prior Work

The vast majority of the large body of available studies that analyzed robot perception and acceptance determinants were based on a simple presentation of a robot by either displaying its photograph or showing a video sequence. Studies in which older participants efficiently interact with real robots are still scarce. Moreover, the main study populations interacting with these robots have included residents of care institutions. Most of these studies have thus been performed in artificial conditions such as in a laboratory or a care home and therefore do not mirror the needs of people who want to live independently [24]. Henceforth, in our study, we targeted older subjects living in the community and their professional caregivers.

Our results, reflecting the opinions and preferences of various stakeholders, contribute to the understanding of human-robot interaction in the studied context and indicate a high general acceptance of a robot in care for older people, expressed by high UNRAQ scores. We previously demonstrated that initial scores were high in the group of older residents of care institutions and increased after interaction with the robot [18]. In the current study, we noted that older people were generally more positive about the robot in care than caregivers. Similarly, more positive opinions of older adults were found in the results of previous studies. Bedaf et al [25] observed that professional caregivers were skeptical about the possibility of convincing older adults to actually use the robot, whereas older participants voiced no concerns toward using a service robot when at home. According to Melkas et al [26], caregivers stressed that care robots presented difficulties with their integration into the workflow and required a substantial amount of time to provide care benefits, which was in a similar way also demonstrated by Bedaf et al [27]. Such views were characteristic of both professional and informal caregivers (who thus might benefit from educational activities related to the introduction of robots in care, adoption of technologies, and development of novel practices to ensure the acceptance and effectiveness of new solutions). One may also consider intergenerational workshops with stakeholders working jointly on detected problems [28-30]. Furthermore, it is worth stressing that the older people in our study scored the UNRAQ statements A6 *(Older adults want to increase their knowledge about the robots to be able to operate them)* and A7 *(The robot should instruct the older person what to do in case of a problem with its operation)* higher than caregivers (*P*=.049 and *P*=.006, respectively).

A systematic review by Vandemeulebroucke et al [31] indicated that in all studies involving interactions with a machine, older people expressed the opinion that the robot would alleviate their loneliness. This review also pointed to the differences in opinions between the participants of studies with and without interaction with the robot. Among the reviewed studies, two involved both older adults and caregivers [32,33]. They also revealed differences between the opinions of stakeholders that should be taken into account when discussing the features of robots to be deployed. In focus group discussions conducted by Bedaf et al [27], older adults also appreciated the permanent availability of a robotic caregiver, which gave them a sense of safety; we noted a need for safety as well, expressed in the scores of statements B1, B6, and B11 of the UNRAQ. Robinson et al [34] also identified the robot’s potential to achieve an increase in neutral and pleasure effects [35,36] and a decrease in depressive symptoms [37,38] and loneliness scores [39,40], which translate into considerable social implications of the use of a robot in care. In our study, the lowest differences between stakeholder groups were observed for assistive functions, which may indicate that the projections of the robot’s use among the studied stakeholders are the most similar in this area, possibly due to its practical, easy-to-imagine dimension.

Our two stakeholder groups showed differences in almost all sociodemographic and declarative data; the only exceptions were living arrangements (alone/with others) and self-reported ease of use of technology. The latter may be viewed as surprising as it is commonly believed that since older people have less contact and experience with modern technologies [41], they are more likely to be afraid of a robot than their younger counterparts, whereas our results do not reflect such a relationship. We observed only an insignificant trend (*P*=.08). Conversely, people who reach retirement age these days generally have good knowledge of modern technologies; it is thus safe to assume that this aspect will influence the intention to use a robot among the “oldest old” only [42]. Additionally, it can be expected that formal caregivers and robots will, in the future, create teams dedicated to caring for older adults [23]. This means that the robot must be independently accepted by both caregivers and older people, although there are significant differences in the characteristics of these groups. Such differences were also observed in other studies that assessed caregivers and older people simultaneously [43]. Moreover, as shown by Papadopoulos et al [44], negative attitudes of caregivers constitute an impediment to the robot’s implementation, while encouragement on behalf of relatives and professionals may have an enabling effect on a robot’s use.

Ethical issues of using humanoid robots in care are rarely analyzed [45]. In our study, the possibility of having control over the robot and the ability to switch it off when needed for privacy reasons was rated higher by older participants than caregivers. This is one of the frequently discussed topics. In earlier studies, both informal and professional caregivers disagreed, mainly due to security concerns, whether older people should be in charge of the robot (which, in turn, might result in programming the robot in a way that would not be in line with the user’s wishes) [25]. Such an approach would certainly undermine the autonomy of the robot’s user. Sharkey and Sharkey [46] also posed the question of who should be held responsible or accountable when a robot responds to the commands of an older person and something goes wrong, possibly resulting in injury or damage. Security of the robot’s use is thus one of the major concerns and the question of security is closely intertwined with ethical issues. Our older participants also voiced this issue by scoring almost all statements from Area D (Ethical issues) significantly higher than caregivers. They also stressed the necessity to prepare the implementation of the robot in a thorough, comprehensive manner, with a clear schedule and sufficiently long presence of a human assistant on-site, which is in line with the abovementioned study stating that “the effect of robots on the lives of the elderly depends on the ways in which they are deployed” [46]. In the study of Carros et al [47], older people did not fear the robot since it was not viewed as a replacement but rather an extension of a (human) caregiver; it is thus legitimate and acceptable that the robot informs the caregiver about noticed problems. Our observations are similar, as statement D5 (*It is acceptable that the robot informs a family member or caregiver about the older person’s behavior/health problems)* was scored high by both groups but higher by the older participants.

### Strengths and Limitations

Our study has some limitations. First, due to its cross-sectional nature—we sampled the perceptions at one particular moment—we have no clues regarding a potential evolution of the opinions of stakeholders along the time axis (which was noted by Fattal et al [48] for some of the assessed assistive, social, and perception items). To some degree, the novelty effect may also influence a short-term interaction with the robot [26]. The second limitation is that we studied the stakeholder groups at different time points in a nonrandomized manner. For future research in this area, it might be beneficial to assemble all participants in one place and time, as Melkas et al [26] observed a partial shift in caregivers’ opinions after witnessing older robot users in action. Nevertheless, we were able to include various stakeholders and obtain a range of observations that can be used for designing robotic interventions in the care of older people.

### Conclusions and Future Directions

The acceptance of the social functions of a robot is related to its perception in all analyzed aspects (Anthropomorphism, Animacy, Likeability, Perceived Intelligence, and Perceived Safety). As for the assistive functions, we observed no significant relationship with Anthropomorphism, which seems to indicate that the expected usefulness of a care robot is not linked to its human-like shape. A thorough analysis of the perception of a robot should thus be an integral part of its design process for both the social and assistive functions.

Given the differences detected in the views of caregivers and older adults in this study and similar observations of other researchers, targeted and properly structured educational and training activities for caregivers and prospective users may provide tangible benefits for the workflow and enable seamless integration of robotic technologies in care provision.

While deploying robots in care for older people, one should take into account both ethical issues and the needs and requirements of all stakeholders involved. Particularly, future users should be able to influence the way the robot’s features and functions are selected and designed. Involving the stakeholders in the cocreation of robotic projects to be implemented can be an inclusive and effective solution.

## Appendix

Multimedia Appendix 1 UNRAQ: Users’ Needs, Requirements and Abilities Questionnaire.

## Abbreviations

GQS: Godspeed Questionnaire Series
UNRAQ: Users’ Needs, Requirements and Abilities Questionnaire

## Data Availability

The data presented in this study are available from the corresponding author upon reasonable request.
