# Supplementary material for: The Effects of Stakeholder Perceptions on the Use of Humanoid Robots in Care for Older Adults: Postinteraction Cross-Sectional Study
Source: J Med Internet Res. 2023 Aug 4;25:e46617. doi: 10.2196/46617 (PMC10439472; doi:10.2196/46617)
Supplement: Multimedia Appendix 1 [file jmir_v25i1e46617_app1.pdf]

UNRAQ

Users' Needs, Requirements and Abilities Questionnaire

<table>
<tr><td colspan="3">Participant ID:</td><td colspan="2">Date</td></tr>
<tr><td rowspan="2">Are you a caregiver?
(please circle)</td><td>YES</td><td rowspan="2">If YES: are you an <u>informal</u> caregiver?
YES NO
If YES, what type:
FAMILY MEMBER
FRIEND
NEIGHBOUR
OTHER (specify):</td><td rowspan="2" colspan="2">...or a <u>formal</u> caregiver:
YES NO
If YES, what type:
SOCIAL WORKER
NURSE
PHYSIOTHERAPIST
OTHER (specify):</td></tr>
<tr><td>NO</td></tr>
</table>

PERSONAL DATA

Date of birth			
Gender	Male	Female	
Education *(number of years)*			
Profession<br>*(if retired, former profession)*			
Background	Technical	Medical	Other
Are you familiar with computers?	YES	NO	Neither Y nor N
Are you familiar with working with technological systems?	YES	NO	Neither Y nor N

Please answer the following questions. There are always 5 possible answers:

I strongly disagree

I partially disagree

I neither agree nor disagree

I partially agree

I strongly agree

**A. INTERACTION WITH THE ROBOT AND TECHNICAL ISSUES**	I strongly disagree	I partially disagree	I neither agree nor disagree	I partially agree	I strongly agree	**Comments**
A1. The robot should be a companion of the older person						
A2. The robot should be an assistant of the older person						
A3. The robot should be a useful device of the older person (something to be used when need, with no other interaction)						
A4. Older adults are prepared to interact with a robot						
A5. Older adults are able to manage with the robot						
A6. Older adults want to increase their knowledge about the robots to be able to operate them						
A7. The robot should instruct the older person what to do in case of problem with its operation						
A8. The robot should be customizable (adjusted to individual user preferences and needs)						
A9. Older adults should be able to choose the functions of the robot they want to use and disable other ones						
A10. If the robot has been switched off by the owner, it should reactivate automatically (after a specific period), so that it is not forgotten in off mode						

B. ASSISTIVE ROLE OF THE ROBOT	I strongly disagree	I partially disagree	I neither agree nor disagree	I partially agree	I strongly agree	Comments
B1. The robot should increase the safety of the older adult's home: e.g. locking doors, detecting leaking gas etc.						
B2. The robot should help the older person to preserve their memory function e.g. by playing memory games with them						
B3. The robot should encourage and guide older adults to perform physical exercises						
B4. The robot should provide advice about a healthy diet						
B5. The robot should monitor the environment (temperature, humidity) and suggest air conditioning adjustment or windows opening						
B6. The robot should measure physiological parameters (blood pressure, heart rate, body temperature) of the elderly person						
B7. The robot should monitor the amount of food and fluid intake of the owner						
B8. The robot should remind older adults about appointments						
B9. The robot should older adults about medication						
B10. The robot should remind about meals times, drinks						
B11. The robot should observe the behaviour of the older person to detect falls or changes due to illness						
B12. The robot should call the centre in case of emergency						
B13. The robot should help the owner to find lost objects (e.g. glasses, keys)						

C. SOCIAL ASPECTS	I strongly disagree	I partially disagree	I neither agree nor disagree	I partially agree	I strongly agree	Comments
C1. The robot could decrease the sense of loneliness and improve the mood of the older person						
C2. The robot could encourage older adults to enhance their contacts with friends						
C3. The robot should initiate contacts with others (calling friends, initiating Skype conversations)						
C4. The robot should have entertainment functions (e.g. gaming partner, reading aloud or playing music function)						
C5. The robot should detect the owner's mood (facial expression)						
C6. The robot should accompany the owner in everyday activities (watching TV, preparing meals)						

D. ETHICAL ISSUES	I strongly disagree	I partially disagree	I neither agree nor disagree	I partially agree	I strongly agree	Comments
D1. The older person should have control over the robot						
D2. The older person should be able to send the robot to its place/docking station and keep it staying there						
D3. It is acceptable that the robot informs a family member or caregiver about the older person's behaviour/health problems						
D4. The older person should be able to switch off the robot in specific situations (friends' visits, privacy reasons etc.)						
D5. It is acceptable that the robot will have much information about the user (social, medical, others)						

CREATIVITY BOX

Please write down all your ideas/suggestions for other functions the robot might have, which could be useful in the everyday life of elderly people.

Give free rein to your imagination. Feel free to report any observations that came to your mind while answering the questionnaire.
